# A case report of an Eurasian jay (*Garrulus glandarius*) attacking an incubating adult and depredating the eggs of the Japanese tit (*Parus minor*)

**DOI:** 10.1002/ece3.9931

**Published:** 2023-03-26

**Authors:** Dake Yin, Xudong Li, Romain Lorrilliere, Zheng Han, Keqin Zhang, Jiangping Yu, Mingju E, Haitao Wang

**Affiliations:** ^1^ School of Life Sciences Changchun Normal University Changchun China; ^2^ Jilin Engineering Laboratory for Avian Ecology and Conservation Genetics, School of Life Sciences Northeast Normal University Changchun China; ^3^ Centre d'Ecologie et des Sciences de la Conservation (CESCO, UMR 7204), Muséum national d'Histoire naturelle, Centre National de la Recherche Scientifique Sorbonne Université Paris France; ^4^ School of Zoological Science Jilin Agricultural Science and Technology University Jilin China; ^5^ Jilin Key Laboratory of Animal Resource Conservation and Utilization Northeast Normal University Changchun China

**Keywords:** hole size, nest predation, secondary hole‐nesting birds, video monitoring

## Abstract

In May 2021, we opportunistically observed one Eurasian jay (*Garrulus glandarius*) attacking an adult incubating Japanese tit (*Parus minor*) and depredating nine tit eggs at a nest box where a woodpecker had greatly enlarged the entrance. After the predation event, the Japanese tits abandoned the nest. We recommend that when using artificial nest boxes to protect hole‐nesting birds, the appropriate entrance size should be proportional to the body size of the target species. This observation gives us a better understanding of the potential predators of secondary hole‐nesting birds.

## Graphical abstract

In May 2021, we incidentally observed one Eurasian jay (*Garrulus glandarius*) attack the incubating Japanese tit (*Parus minor*) adult bird and depredated nine tit eggs at a nest box where a woodpecker had greatly enlarged the entrance. After the predation event, the Japanese tits abandoned the nest. This discovery gives us a better understanding of the potential predators of the secondary hole‐nesting birds.

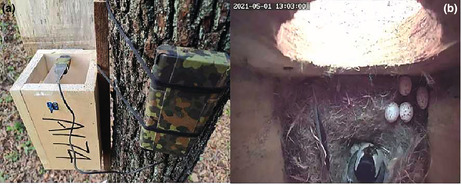

## 1 INTRODUCTION

For hole‐nesting birds, entrance size is a critical feature of nest‐site suitability (Le Roux et al., 2016). Large entrances make cavities less attractive to small birds because cavities with smaller entrances can provide increased protection against predators and competitors (Valera et al., 2019, but see Juškaitis, 2021). Previous studies have found that smaller entrances are adaptive because they can prevent many predators from passing through the entrance and accessing nest contents (Markovec & Visotsky, 1993; Walankiewicz, 1991). Besides adequate entrance size, the nest box's depth is also considered one of the factors affecting bird reproduction because shallow nests become more prone to depredation (Nilsson, 1984). Therefore, a secure cavity should have an entrance proportional to the body size of the hole occupant (Le Roux et al., 2016; Wesołowski, 2002) and have a sufficient depth not to be easily damaged by predators (Nilsson, 1984; Wesołowski, 2002).

Although cavity nests offer protection from many predators, nest predation is still the main reason for the reproductive failure of hole‐nesting birds (Lima, 2009; Martin, 1993; Martin & Li, 1992). The species and type of predator depends on the geographical area and habitat type (Shen et al., 2022). Therefore, the mode of predation and selective forces on nest sites may vary (Czeszczewik, 2004; Picman & Schriml, 1994). For example, snakes are highly specialized egg‐eating (e.g., Gartner & Greene, 2008) or nestlings in the nest and raptors may prefer to attack the nestlings from outside the nest entrance using their beaks or legs (Barnett et al., 2013; Cox, Thompson, et al., 2012; Suzuki & Ueda, 2013). Due to the limitation of entrance size, the predators of hole‐nesting birds mainly include rodents, mustelids, woodpeckers, and snakes (Wesołowski, 2002), whereas avian predation on cavities is rare.

Knowing more about the identity of predators and their mode of predation helps us to predict the prevalence of nest loss (Cox, Pruett, et al., 2012; Cox, Thompson, et al., 2012) and to better understand ecological interactions with a goal of conservation (Chalfoun et al., 2002; Lima, 2002; Schmidt, 1999). It also helps us to understand the selective pressures affecting parental and offspring antipredator strategies (Ibáñez‐Álamo et al., 2015). However, due to the frequency and discreetness of natural predation events, it is not easy to directly witness the predation process. Researchers usually speculate on potential predators based on the remains of abandoned nests (Williams & Wood, 2002), which may result in the actual nest predators being different from the assumed predators (Peterson et al., 2004). Video cameras are widely used to monitor the behavior of breeding bird and sometimes to record. For example, the video recording showed the process of multiple host individuals of the Oriental reed warbler (*Acrocephalus orientalis*) mobbing and attacking a female common cuckoo (*Cuculus canorus*) in the field (Zhao et al., 2022). In addition, the video cameras are able to accurately record nest predation cases of birds (Ball & Bayne, 2012; Bolton et al., 2007). Here, we report a case of predation by the Eurasian jay (*Garrulus glandarius*) on a Japanese tit (*Parus minor*) nest that we observed while monitoring.

## 2 METHODS

**FIGURE 1:**
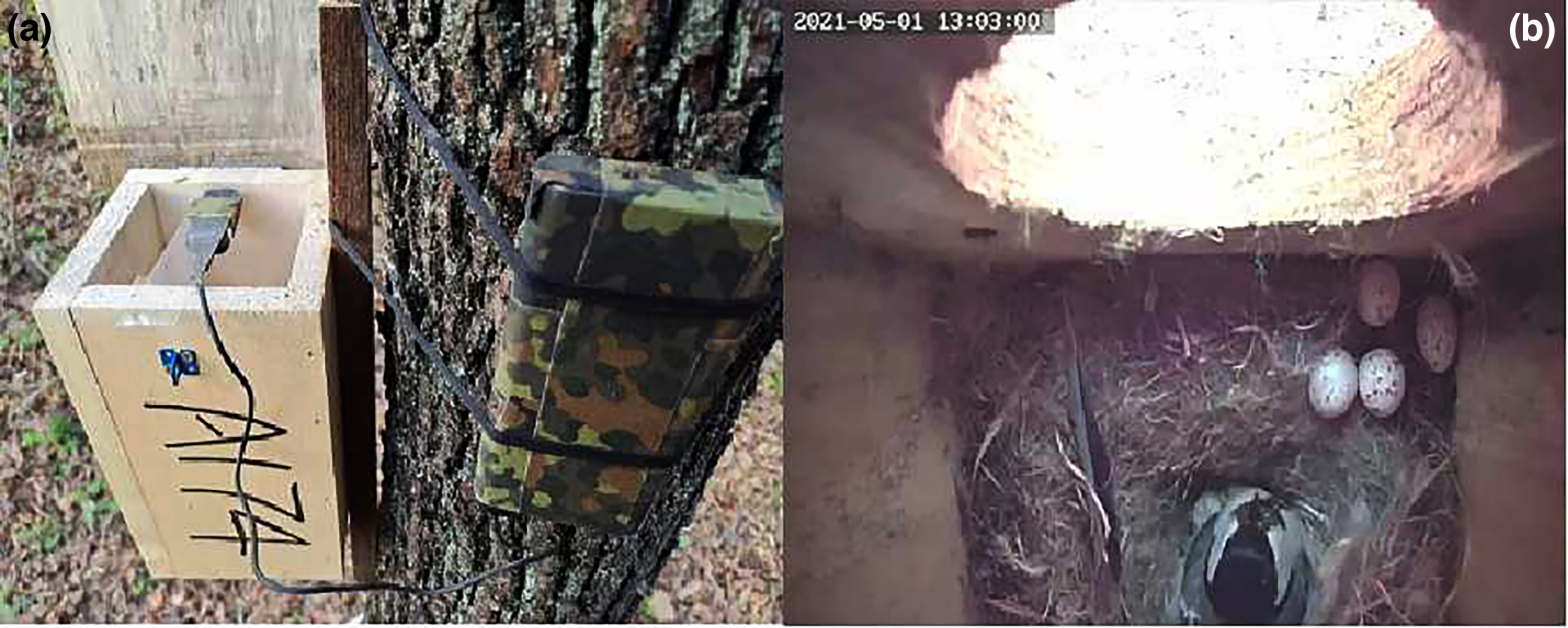
(a) Nest box monitoring setup showing a mini digital camera affixed under the roof of the box and connected with a cable to the external battery pack. (b) The Japanese tit returned to the nest to continue incubating after nest predation.

The study area is located at Zuojia Nature Reserve (44°1′‐45°0″ N, 126°0′‐126°8″ E) in Jilin Province, Northeastern China. Since 2004, we have attached the artificial nest boxes to trees approximately 3–4 m above the ground. The entrance hole size of artificial nest boxes in our study area was 4.5–6.5 cm (Yu et al., 2021). The nest boxes have these dimensions: The height is 25 cm, on 12 cm × 12 cm. In 2021 from 27 April to 9 May, we used a mini digital video camera (CTS‐K6; Chuangtianshi Technology Co., Ltd.) to monitor the incubating behaviors of Japanese tits in 22 nest boxes from 08:00 a.m. to 17:00 p.m. (GMT + 8). We fixed the camera on the inner lid of the box and adjusted the camera's angle to ensure the recording of the nest cup and entrance hole (Figure 1). The entrance hole of one of the nest boxes had been enlarged by a woodpecker (*Dendrocopos* spp.) pecking at it, such that the entrance to this box was 7.5 cm in diameter, and the depth from the entrance to the bottom was about 14.0 cm. However, we did not measure the thickness of Japanese tits' nesting material in the nest box.

## 3 RESULTS

On May 1, 2021, we opportunistically observed one Eurasian jay attacking an adult incubating Japanese tit and depredating nine tit eggs at a nest box with a 7.5‐cm entrance hole through the video cameras for the first time.

The video showed a female Japanese tit incubating her 12 eggs as usual. An abnormal noise was heard outside the nest box at 12:32:44. Then, the incubating female Japanese tit showed a hissing behavior. At 12:32:48, the Eurasian jay appeared at the hole entrance for the first time and put its head into the nest box to attack the tit, who made a second hissing behavior. In order to avoid predation, the female tit was forced to the corner of the nest box. However, the jay made a second attack and tried to catch the adult bird, making the tit lose some feathers (Video S1). At 12:32:50, both the jay and the tit disappeared from view. Looking at the video in slow motion, we can see the jay extracting the tit from the box by grabbing it at the leg. From 12:33:53 to 13:49:35, the jay repeatedly returned to the nest box and successfully fetched nine tit eggs (Video S2). Simultaneously, an adult Japanese tit entered the nest box several times to incubate eggs (from 13:02:33 to 14:52:03; Video S3, Figure 1b) when the jay was absent. It is worth noting that the jay tried nine times for the last predation, and finally, only one egg was eaten. At 5 p.m., after retrieving the video camera and replaying the video, we found the nest predation fact and assured Eurasian jay was the nest predator. From 05:22:05 to 18:19:58 on May 2, 2021, we monitored this nest through the digital video camera. At 09:08:05, a Eurasian jay appeared again and tried to prey on the remaining eggs in this nest but failed (Video S4). One adult Japanese tit flew back to incubate from 11:08:19 to 11:35:09. No bird was recorded after that. We confirmed that the Japanese tits had abandoned the nest and suspended video monitoring of this nest box.

## 4 DISCUSSION

We incidentally observed one Eurasian jay attacked incubating tit and successfully depredated nine tit eggs in this study. The appropriate entrance size should be proportional to the body size of the target species, and RSPB recommends a hole diameter of 28 mm for the great tit nest box (see details in https://www.rspb.org.uk/). If the hole size were this small, we believe that depredation by the jay could not have occurred. However, the hole sizes selected by Japanese tits in our study area ranged from 2.8 cm to 7.5 cm. For example, Ma et al. (2015) found that 12 pairs of Japanese tit occupied old woodpecker holes with an entrance diameter of 5–7 cm in 2014. In this case, Japanese tits occupied a nest box with a 7.5 cm hole entrance, which allowed Eurasian jays to predation. Considering that jays are the conventional nest predators of some open‐nesting birds (Weidinger, 2009), we suggested that Eurasian jays are opportunistic nest predators of cavity‐nesting birds in our study area.

The jay repeatedly returned to the nest box during the predation process. Here, we cannot determine whether the jay that appears multiple times in the video was the same individual. However, the Eurasian jay appeared again the next day for predation, supporting that the jay has spatial memory and search image of the food source (Cheke et al., 2011; Cramp, 1994; Sonerud, 1985). As the nest box was locked, the jay could not open the nest box during the whole process of predation and could only attack adult bird and depredate eggs through the hole. Therefore, smaller entrances are still important to prevent predation events.

The video showed the jay pulling the adult tit from the box by its leg but we cannot be certain that it finally killed the bird. Nevertheless, previous studies suggest there is a high danger for birds in cavity nests because there is only one route to escape during a predator attack (Low et al., 2010). Since only female tits are known to incubate (Ding et al., 2014), the female may have returned to the nest box but subsequent incubation was erratic (Figure 1b) and it was eventually abandoned.

Tits have evolved a variety of nest defense behaviors to prevent their offspring and themselves from being harmed by predators, such as alarm calls, mobbing, and attacks (Shen et al., 2022; Yu et al., 2021). When a predator or human approaches the nest of tits, the incubating females perform hissing display that mimics the inhalation hiss of a viper or another snake to deter the intruders (Koosa & Tilgar, 2016; Moller, Flensted‐Jensen, et al., 2021; Moller, Gil, et al., 2021). However, hissing calls are not always successful enough to deter the predator away, because the efficiency of hissing calls relies on the fact that predators respond to hisses (Wickler, 2013). For example, the rate of nest predation of Japanese tits, willow tits (*Poecile montanus*), and coal tits (*Periparus ater*) that hissing and nonhissing is similar in Saihanba National Forest Park in China (Zhang et al., 2020). Similarly, we also found that the hissing calls of Japanese tit did not work for the jay in our observation.

We have occasionally observed common chipmunks (*Tamias sibiricus*) and snakes preying on eggs or chicks in nest boxes through video surveillance several times. It was the first time we observed a jay attack an adult tit and prey on eggs in a nest box in our study areas. In addition, this observation gives us a better understanding of the potential predators of the secondary hole‐nesting birds. Our observation confirms that continuous video monitoring of nests is a reliable and practical method to record predators and (anti‐) predation behaviors (Benson et al., 2010; Weidinger, 2008, 2009).

## AUTHOR CONTRIBUTIONS

**Dake Yin:** Investigation (equal); visualization (equal); writing – original draft (lead). **Xudong Li:** Investigation (equal); visualization (equal). **Romain Lorrilliere:** Writing – review and editing (equal). **Zheng Han:** Visualization (equal); writing – review and editing (equal). **Keqin Zhang:** Funding acquisition (equal); investigation (equal). **Jiangping Yu:** Writing – review and editing (equal). **Mingju E:** Funding acquisition (equal); writing – review and editing (equal). **Haitao Wang:** Funding acquisition (equal); resources (lead).

## FUNDING INFORMATION

This work was supported by the Projects of Jilin Province Science and Technology Development Plan YDZJ202201ZYTS665 to ME, Scientific Research Fund of Jilin Provincial Education Department JJKH20220835KJ and the Natural Science Foundation of Changchun Normal University 202010205057 to ME. National Natural Science Foundation of China 31971402 to HW, 31870368 to KZ.

## CONFLICT OF INTEREST STATEMENT

None declared.

## Supporting information


Video S1.

Video S2.

Video S3.

Video S4.

## Data Availability

Supplementary material “Video S1, S2, S3, S4” at the Dryad Digital Repository: https://doi.org/10.5061/dryad.zcrjdfnhs.
